# A snapshot of the electrochemical reaction layer by using 3 dimensionally resolved fluorescence mapping†
†Electronic supplementary information (ESI) available: Additional figures displaying cyclic voltammetry, fluorescence spectroscopy, experimental setup, *in situ* evolution of fluorescence intensity and chronoamperometry data. See DOI: 10.1039/c8sc02011f


**DOI:** 10.1039/c8sc02011f

**Published:** 2018-07-16

**Authors:** Anne de Poulpiquet, Bertrand Goudeau, Patrick Garrigue, Neso Sojic, Stéphane Arbault, Thomas Doneux, Laurent Bouffier

**Affiliations:** a Univ. Bordeaux , CNRS , Bordeaux INP , ISM , UMR 5255 , F-33400 Talence , France . Email: laurent.bouffier@enscbp.fr; b CHANI , Faculté des Sciences , Université libre de Bruxelles (ULB) , CP 255 , B-1050 Bruxelles , Belgium . Email: tdoneux@ulb.ac.be

## Abstract

The coupling between electrochemistry and fluorescence confocal laser scanning microscopy (FCLSM) allows deciphering the electrochemical and/or redox reactivity of electroactive fluorophores. This is demonstrated with phenoxazine electrofluorogenic species frequently used in bioassays by mapping the variation of fluorescence intensity with respect to the distance from the electrode. The electrochemical conversion of resorufin dye (RF) to non-fluorescent dihydroresorufin (DH) leads to a sharp decrease of the fluorescence signal in the vicinity of the electrode. In contrast, the direct reduction of resazurin (RZ) to DH leads to an unexpected maximum fluorescence intensity localized further away from the surface. This observation indicates that the initial electron transfer (heterogeneous) is followed by a chemical comproportionation step (homogeneous), leading to the formation of RF within the diffusion layer with a characteristic concentration profile. Therefore, *in situ* FCLSM affords a direct way to monitor such chemical reactivity in space and to decipher a new redox pathway that cannot be resolved solely by electrochemical means.

## Abstract 2

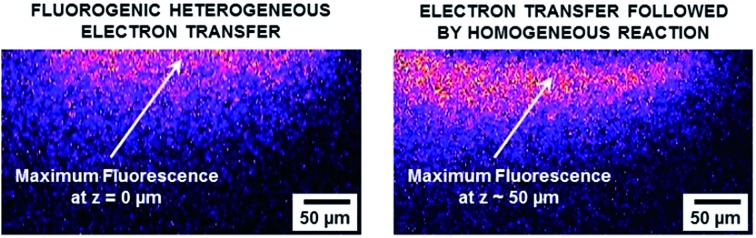
Fluorescence confocal laser scanning microscopy under electrochemical control allows imaging of various reaction layers revealing heterogeneous *versus* homogeneous reactions.

## Introduction

Electrochemistry (EC) offers a range of experimental methods to investigate reactions that involve electron transfer (ET). Typical time-resolved measurements provide thermodynamic as well as kinetic information. Electrochemical techniques have therefore been implemented to elucidate various organic or inorganic reaction mechanisms.^1^–^4^ From a molecular point of view, EC gives access not only to the reactivity but also to the transport properties of the chemical species involved. In fact, EC allows probing the concentration profiles in the diffusion layer, though in an indirect way. However, it does not always give access to the full distribution of individual chemical species, especially for unstable electrogenerated species that react further in the diffusion layer. Comproportionation is a typical case in this respect: in many situations (*i.e.* fast reaction and similar diffusivity of all species), the occurrence of comproportionation has only a small effect on the shape of either the voltammetric or the chronoamperometric response.

Gaining an additional level of information during electrochemical reactions thus requires the coupling of EC to other techniques. For example, a local-probe approach was proposed that involves positioning a microelectrode perpendicular to the working macroelectrode surface enabling the probing of the local concentration of chemical species within the diffusion layer.^5^ This method was employed to elucidate reaction mechanisms, especially in the case of tetracyanoquinodimethane (TCNQ) which undergoes a comproportionation reaction with its two-electron reduction product.^6^ In a far-field approach, access to the local chemical composition of the electrolyte is also possible by optical methods. Amatore *et al.* used confocal Raman micro-spectroscopy to study the spatial distribution of TCNQ˙^–^ produced by comproportionation, thus providing unambiguous spectral evidence of such chemical reactivity.^6^–^8^ However, these measurements required a quite high concentration range (typically mM) due to the intrinsic low sensitivity of the Raman scattering signal whose intensity is limited by poor cross-sections (Raman cross section *α* 1/*λ*^4^).

**Scheme 1:**
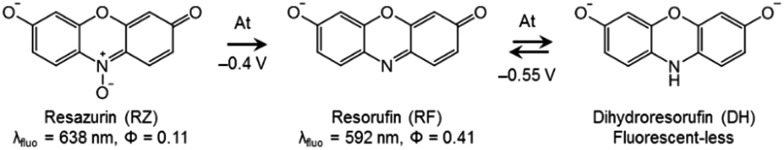
Structures and properties of the studied dyes.

Alternatively, fluorescence imaging is a versatile and highly sensitive technique that allows getting spatially resolved information and enables quantitative analysis.^9^ Fluorescence microscopy coupled to EC, proposed first by Engstrom and coworkers in the '90s,^10^,^11^ is an efficient tool to image diffusion layers in either 2D (profile view or lateral cross section) or 3D (stacks of cross sections).^12^,^13^ Fluorescence confocal laser scanning microscopy (FCLSM) allows recording of fluorescence in a micrometer-sized volume. In contrast to wide-field microscopy, the fluorescence signal is collected from an optical slice with very low field depth at a given focal plane.^14^ The *in situ* coupling of this technique with EC (EC-FCLSM) affords 3D fluorescence imaging of any electrochemical reaction that modulates the concentration of a fluorescent species. EC-FCLSM was employed to image pH gradients induced by proton-coupled electrochemical reactions at a microelectrode when using pH-sensitive fluorescent dyes.^15^ Moreover, the fluorescence signal was demonstrated to be quantitative in comparison with numerical simulations.^16^ More recently, EC-FCLSM was used to image the steady-state diffusion profile during the electrochemical reduction of resazurin (RZ) to resorufin (RF).^12^ Because concentration profiles can be reconstructed from fluorescence images, EC-FCLSM is a powerful approach to elucidate molecular mechanisms. For example, it allowed the determination of the number of electrons involved in the electrochemical reduction of O_2_,^16^ or deciphering the oxidative mechanism responsible for Amplex® Red fluorescence by *in situ* formation of RF.^17^ In this work, we demonstrate that EC-FCLSM is well-suited to probe the chemical reactivity inside the diffusion layer. For that, we selected again RZ and RF dyes which are very important molecular probes used in many bioassays (Scheme 1). We report direct visual evidence of a comproportionation reaction between the fluorescence-less dihydroresorufin (DH) and the oxidized weakly fluorescent RZ species, leading to the highly fluorescent RF. To the best of our knowledge, this is the first time that such a homogeneous reaction is evidenced by *in situ* FCLSM rather than simple heterogeneous ET processes.

## Results and discussion

DH/RZ/RF are very appropriate dyes from a spectroelectrochemical point of view because the fluorescence of these species is directly influenced by the corresponding redox state, thus allowing electrochemically induced fluorescence modulation. These phenoxazines are indeed becoming increasingly popular in coupled EC fluorescence studies.^18^–^20^ The oxidized form RZ is only fairly fluorescent (*λ* = 638 nm, *φ* = 0.11).^21^ RF, which is electrogenerated through a two-electron irreversible reduction of RZ, is highly fluorescent (*λ* = 592 nm, *φ* = 0.41).^21^ Also, RF can be further reduced reversibly, through another 2-electron process, to the non-fluorescent DH. Therefore, the appearance and disappearance of fluorescence can be readily achieved *in situ* under potential control. The results of cyclic voltammetry (CV) experiments are presented in Fig. S1.† RZ is typically reduced to RF at –0.4 V *vs.* Ag/AgCl (at pH 10) whereas the reversible reduction of RF to DH occurs at –0.55 V.

Fluorescence spectroscopy was first employed to determine the optimal concentrations of the fluorescent species for further EC-FCLSM experiments. RZ and RF fluorescence spectra were recorded at increasing concentrations in the range 0–500 μM (Fig. S2a and S2c†). The typical shape and characteristic wavelengths of both RZ and RF spectra perfectly match with literature data.^12^ The fluorescence intensity at the wavelength of maximum emission exhibits a linear variation with fluorophore concentration up to 100 μM (Fig. S2b and S2d†). Therefore, further experiments were conducted at a fluorophore concentration of 100 μM in order to maximize the signal intensity but can possibly be conducted at a lower concentration. Typically the low μM range is suitable to perform EC-FCLSM as demonstrated previously when using a 5 μM fluorophore concentration.^16^ In the case of the present study, 100 μM was found to be a good compromise in order to record and analyse simultaneously both electrochemical and fluorescence microscopy data. The fluorescence was also recorded during the course of classic spectroelectrochemical experiments (Fig. S3†). The fluorescence is collected in the whole electrolytic volume exposed to the excitation beam (∼100 μL). The cell initially contained a RZ solution and the potential of the mesh working electrode was first set at –0.45 V (*i.e.* just after the first reduction peak). The fluorescence consistently increased with time exhibiting a gentle slope without reaching a plateau in the timeframe of the experiment (*t* >1000 s). This time trace is intrinsically linked to the thin-layer geometry of the electrolytic cell. If instead of –0.45 V the potential was set at –0.6 V, the fluorescence level did increase much faster (*i.e.* steeper slope), which is counter-intuitive since at this potential only the non-fluorescent DH should be thermodynamically formed. Also, the fluorescence intensity associated with RF formation is even higher at this potential. Such an observation could only be explained by considering an additional chemical reaction that regenerates RF dye under the tested experimental conditions.

**Fig. 1:**
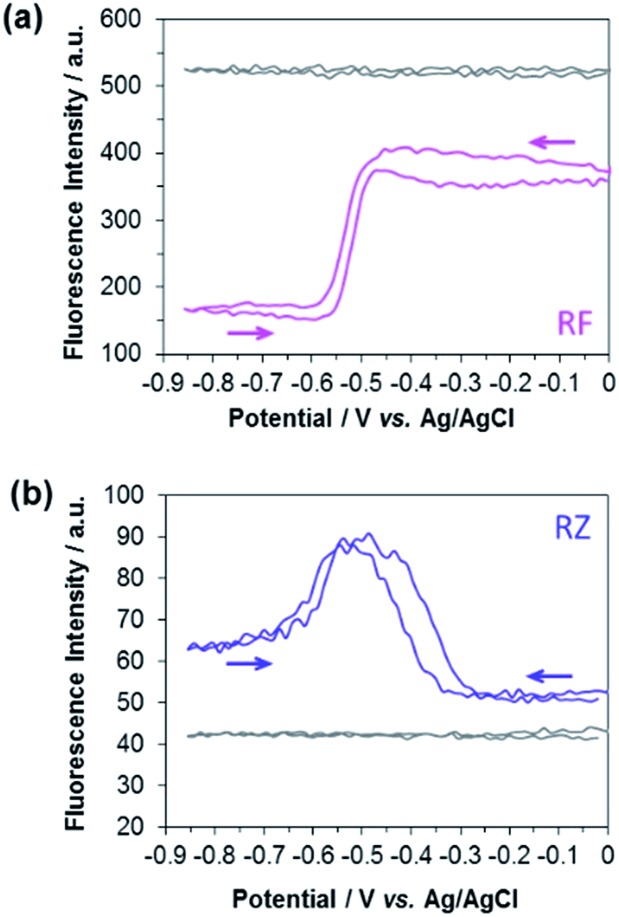
Evolution of the fluorescence intensity at the electrode surface (ROI-1) during a cyclic voltammetry experiment recorded in a 25 mM carbonate buffer solution (pH 10) containing 100 μM (a) RF (pink line) and (b) RZ (blue line). Grey lines indicate the variation of bulk fluorescence away from the electrode surface (ROI-2).

The experimental EC-FCLSM setup was already described in previous reports.^12^,^22^ Briefly, the electrochemical cell is placed above the objective of an inverted fluorescence confocal microscope (Scheme S1†). The working electrode is positioned in front of the objective, and is separated from the bottom of the electrochemical cell by a few millimetres. This setup offers the possibility of recording images at the electrode surface and also at different *z*-positions within the solution. The electrode|solution interface was readily localized in reflection mode with a red laser (*λ* = 633 nm). The depth of field was adjusted by using a pinhole value corresponding to 1 Airy Unit. This setting is very classic in CLSM as it offers a good compromise to enable a reasonable fluorescence signal without impeding axial (*z*) resolution. Fluorescence was collected between 570 and 610 nm in two regions of interest (ROIs) of 50 × 50 μm^2^. The first ROI (ROI-1) is defined on the electrode surface while the second one (ROI-2) is located in the bulk far from the electrode surface (Fig. S4†). In each case, the evolution of the mean fluorescence intensity in each ROI is studied as a function of the applied potential. A series of images were acquired *in situ* during voltammetric or chronoamperometric experiments performed in a solution containing either RZ or RF with the fluorescence being recorded from a given confocal volume. Fig. 1 shows the electrofluorograms recorded in the two ROIs with 100 μM RF (Fig. 1a) or RZ (Fig. 1b). The corresponding time-dependent fluorescence evolution profiles are given in Fig. S5.† As expected, the fluorescence intensity does not change with potential in ROI-2, being low in the RZ solution and much higher in the RF solution (grey lines). The fluorescence intensity at the electrode surface is slightly different from the bulk value in the absence of electrochemical reaction. This may be due to the presence of the electrode inside the probed confocal volume (*i.e.* masking effect) compared to the bulk where the corresponding volume is fully filled with the solution. The working electrode potential was scanned from 0 V down to –0.85 V and back, like in the conventional CV experiment shown in Fig. S1.† In the presence of RF (Fig. 1a), the fluorescence in ROI-1 is high and decreases sharply at around –0.5 V in the forward scan because of the reduction of RF to DH. Fluorescence consequently remains constant as long as the electrode potential is negative enough to drive RF reduction. Afterwards, the signal increases again at –0.5 V in the reverse scan, when DH is re-oxidized to RF. When the electrolyte solution contains RZ (Fig. 1b), the initial fluorescence intensity is very low until a marked fluorescence rise starting at around –0.3 V is observed, which is in full agreement with the formation of RF. Then, the fluorescence intensity starts to decrease at around –0.5 V, which is consistent with the subsequent reduction of RF to DH. However, the fluorescence level does not decrease back to its initial value. In the reverse scan, DH is re-oxidized to RF and the fluorescence consistently increases again and reaches the same maximum as during the forward scan. At higher potentials, fluorescence finally decreases back to the initial intensity because RF molecules diffuse away from the electrode surface. A similar fluorescence variation is observed when consecutive potential steps are applied instead of a potential sweep (Fig. S6†), with the fluorescence at –0.8 V being significantly higher than that at –0.35 V, even though both RZ and RF are reduced to DH at this very negative potential value. Such an unexpected behaviour has never been reported and we hypothesized the occurrence of a comproportionation reaction between RZ present in the bulk and DH formed at the electrode to produce RF fluorophore according to

1 DH + RZ → 2RF

The corresponding Gibbs energy Δ*_r_G* of this hypothetical reaction between RZ and DH can be estimated from the apparent formal potentials of the RZ/RF and RF/DH redox couples, respectively:2
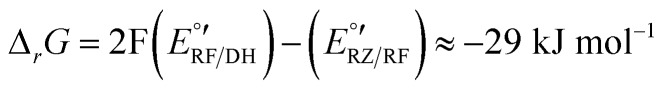



Reaction (1) is indeed very exergonic, meaning that the coexistence of DH and RZ is thermodynamically unfavourable.

**Fig. 2:**
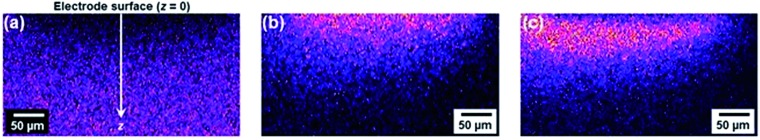
Fluorescence intensity mapping images along the *z*-direction collected by EC-FCLSM. Data recorded in a 25 mM carbonate buffer solution (pH 10) containing 100 μM (a) RF or (b and c) RZ. Potential steps: –0.8 V (a), –0.55 V (b) and –0.75 V (c), respectively.

A comproportionation is a homogeneous reaction that should markedly affect the concentration profiles of all the involved species.^6^ In the next set of experiments, EC-FCLSM was therefore performed in order to map the concentration profile of RF in the volume localized in the vicinity of the electrode surface, after applying various potential steps to the working electrode immersed in either a 100 μM RF or RZ solution. In the steady state, *i.e.* once the current had reached a constant value (Fig. S7†), the confocal plane was moved along the axial direction (*z*) perpendicular to the electrode surface (*x*–*y* plane). Following the evolution of the fluorescence intensity with the distance from the electrode surface allows probing the whole volume of the diffusion layer and rebuilding the 3D concentration profile of the fluorescent species. As before, the mean RF fluorescence intensity was measured in two ROIs for each axial position, and was plotted as a function of the distance *z* from the electrode surface (as a convention, *z* = 0 at the electrode surface). In each case the potential was applied stepwise to the same electrode, after a sufficient relaxation time, whereas all other parameters were kept constant (laser beam intensity, *z* origin, *z* step, *etc.*). Representative orthogonal views of these *z*-stacks are presented in Fig. 2. For RF reduction to DH at –0.8 V, the mapping reveals a homogeneous fluorescence in the bulk which is suppressed at the electrode surface as the electrogenerated DH is not fluorescent (Fig. 2a). The exact opposite trend is observed when RZ is reduced to RF at a mild potential value of –0.55 V with a marked fluorescence in the vicinity of the electrode whereas the bulk of the solution does not fluoresce (Fig. 2b). The thickness of the diffusion layer is consistent with previous data collected with the same electrode size,^12^ and the maximum fluorescence level is directly localized at the electrode surface (*i.e. z* = 0). If a more negative potential is applied in the same solution (–0.75 V), the same features are observed except that the fluorescence intensity is not anymore maximum at *z* = 0 but is visible further away from the electrode surface, typically at several tens of micrometres (Fig. 2c). Both fluorescence intensity mapping images presented in Fig. 2b and c were solely collected from the RF fluorescence signal, avoiding any possible interference from RZ. This is directly due to the collection mode that involves a spectral selector module which enables selection of the wavelength bandwidth of the collected fluorescence. It was set in the range 570–610 nm, thus corresponding to the RF maximum fluorescence whereas RZ fluorescence is nil in that region as its emission in centred at a lower energy (*λ* = 638 nm). It is also noteworthy that this spectral selector module enables a reconstruction of a fluorescence spectrum by varying the collection bandwidth. It was employed previously to confirm that the fluorescence signal collected definitely arises from the electrogeneration of RF.^12^

**Fig. 3:**
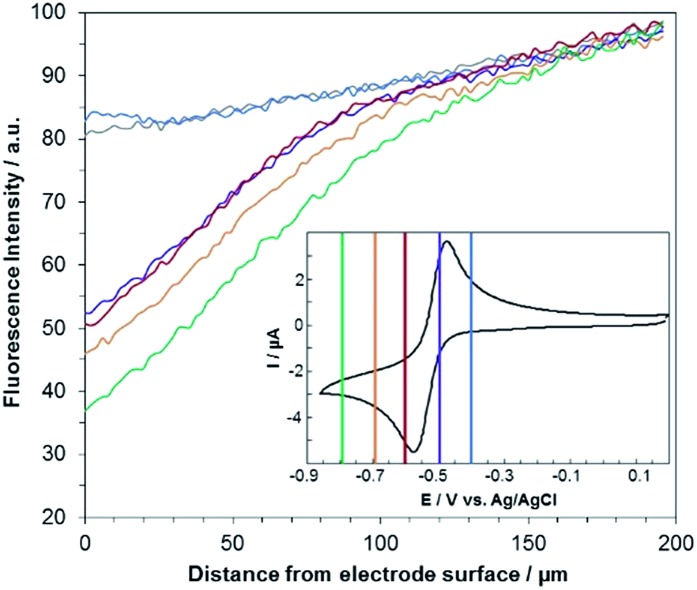
Steady state fluorescence profiles recorded at the electrode surface (ROI-1) when various potential steps are applied in a 25 mM carbonate buffer solution (pH 10) containing 100 μM RF. Potentials: –0.4 V (blue curve), –0.5 V (violet), –0.6 V (red), –0.7 V (orange) and –0.8 V (green) with a *z*-step of 2 μm. The grey line indicates the fluorescence in the bulk of the solution (ROI-2). Inset: cyclic voltammetry of RF with coloured lines indicating the potential steps.

**Fig. 4:**
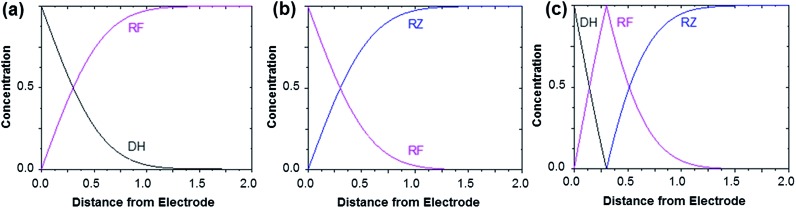
Concentration profiles obtained after applying a sufficient potential step in order to reduce RF to DH (a), to reduce RZ to RF (b) and to directly convert RZ to DH (c). In the latter case, a comproportionation occurs between DH and RZ to form RF. The horizontal axis is given in arbitrary units (dimensionless). These theoretical profiles are calculated for semi-infinite linear diffusion at a planar disk electrode, but the morphology of the curves (monotonic or peak-shaped) is qualitatively similar for other electrode geometries.

Fig. 3 shows the evolution of the fluorescence intensity along the *z*-axis when different potentials are applied to the working electrode, with RF in the electrolyte solution. A tiny fluorescence variation is seen in the bulk (ROI-2, grey line) and in ROI-1 when a potential above the reduction peak is applied (blue line). At more negative potentials, the fluorescence is stable in the bulk whereas a fluorescence drop is clearly observed at the electrode surface. This corresponds to the electrochemical formation of DH from RF, which causes fluorescence extinction. The fluorescence intensity increases with distance from the electrode surface until it reaches its maximum at approximately 150 μm, which is consistent with the DH concentration gradient in the diffusion layer. This expected behaviour is indeed the signature that no other chemical transformation occurs in solution following the ET at the electrode surface under these conditions (Fig. 4a).

**Fig. 5:**
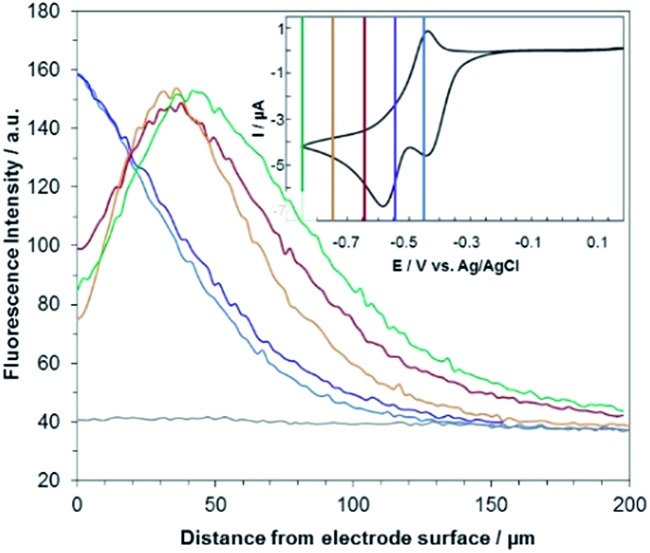
Steady state fluorescence profiles recorded at the electrode surface (ROI-1) when various potential steps are applied in a 25 mM carbonate buffer solution (pH 10) containing 100 μM RZ. Potentials: –0.45 V (blue curve), –0.55 V (violet), –0.65 V (red), –0.75 V (orange) and –0.85 V (green) with a *z*-step of 2 μm. The grey line indicates the fluorescence in the bulk of the solution (ROI-2). Inset: cyclic voltammetry of RZ with coloured lines indicating the potential steps.

A comparable set of experiments performed with a 100 μM RZ solution is given in Fig. 5. Again, the fluorescence recorded in the bulk does not vary with the distance from the electrode surface (grey line in Fig. 5). Similarly, the current intensity increases slightly when more negative potentials are applied in chronoamperometry experiments (Fig. S8†). This simply indicates that a larger charge is injected during the surface-confined ET reaction with increasing overpotentials. However, it does not give any indication of the fate of the species electrogenerated at the electrode. In contrast, the fluorescence profiles feature two distinct behaviours depending on the applied potential. When the potential value is set to be less negative than the second cathodic peak (Fig. 2b and 5, blue/violet lines), fluorescence is maximal at the electrode surface and decreases with increasing distance. Such potentials drive the electrochemical conversion of RZ to RF which is consistent with previous data.^12^ RF is electrogenerated at the electrode and then diffuses towards the bulk, which leads to a concentration gradient of RF.

The corresponding qualitative concentration profile is given in Fig. 4b. It is noteworthy that the theoretical profiles provided in Fig. 4 are not quantitative and are therefore presented in dimensionless units. Nevertheless, the blue or violet lines in Fig. 5 are self-consistent with the highest RF concentration localized at *z* = 0 and a progressive decrease towards the bulk of the solution, which is typical of a monotonic concentration profile.

At lower potentials, the maximum fluorescence intensity is not detected at *z* = 0 but at a focal plane situated inside the diffusion layer, approximately 50 μm away from the electrode surface. Such “bell-shape” fluorescence profiles (and hence concentration profiles) are direct evidence of the comproportionation reaction taking place in solution. Indeed, in this potential range, the 4e– reduction of RZ to DH occurs at the electrode, meaning that RF formation is avoided under these conditions for thermodynamic reasons. The decrease of fluorescence intensity at the electrode surface compared to the experiments performed at higher potentials is therefore predictable as DH is not fluorescent. However, the increase of fluorescence when scanning in the *z*-direction indicates that RF is formed in solution rather than at the electrode|solution interface, by redox reaction between DH diffusing away from the electrode and RZ diffusing towards it. The maximum intensity is similar to that recorded when RF is directly formed at the electrode from RZ which is again in agreement with the concentration profiles expected for a fast comproportionation reaction (Fig. 4c), one notable difference being that the experimental fluorescence at the electrode is higher than expected. This is likely due to the fact that the confocal volume is not infinitely thin. Also, the theoretical profiles were calculated from existing analytical solutions derived for semi-infinite linear diffusion at a planar disk electrode and assuming an infinitely fast comproportionation.^23^ This comproportionation reaction, which is indeed predictable by simple thermodynamic considerations, is not detectable in conventional EC but is unambiguously evidenced when using EC-FCLSM. The electrogeneration of DH from a RZ solution leads to the formation of RF and consequently to fluorescence generation. The corresponding concentration profiles that take into account the comproportionation reaction are given in Fig. 4c. In this case, one could expect that fluorescence is nil at *z* = 0 since RZ molecules are fully consumed at the electrode surface. In contrast, DH concentration is maximal at the surface, but a comproportionation could not be observed at this position since there are no RZ molecules. When the electrogenerated DH molecules leave the electrode toward the bulk of the solution, they might ultimately encounter RZ molecules and react according to the comproportionation. In that case, the maximal fluorescence plane should be situated approximately in the middle of the diffusion layer if both species exhibit a comparable diffusion coefficient. A slight deviation from that position might indicate that the electrochemical reaction may be faster than the chemical one or that the location of the counter electrode might affect the diffusion of the involved chemical species. Nevertheless, EC-FCLSM proves to be a very effective tool here to probe such chemical reactivity occurring inside the diffusion layer. Also, thanks to the high sensitivity of fluorescence, the proposed 3 dimensionally resolved fluorescence mapping could be potentially conducted down to a very low dye concentration. Even if the limit of detection has not been investigated in the present study, a statistically significant variation of fluorescence upon electrochemical conversion can be estimated in the low micromolar range. Finally, working at low fluorophore concentration allows minimization of the inner filter effect generally observed in absorbing solutions. Such undesirable effects were for instance reported in FCLSM experiments involving millimolar solutions of Ru(bpy)_3_^2+^ used as a model dye,^24^ and were overcome by the use of a transparent electrode and back-illumination. The present approach is more versatile, since any type of electrode can be employed.

## Experimental

### Chemicals

Experiments were performed in 25 mM sodium carbonate (Na_2_CO_3_, NaHCO_3_) buffer (pH 10) prepared with ultrapure water (resistivity 18.2 MΩ cm at 25 °C). Na_2_CO_3_ (purity 98%) was purchased from Alfa Aesar. NaHCO_3_ (purity ≥95%), resazurin sodium salt (BioReagent grade) and resorufin (dye content 95%) were purchased from Sigma-Aldrich. All reagents were used without further purification.

### Electrochemical experiments

Electrochemical experiments were performed with a PGSTAT potentiostat from Metrohm. The working electrode was a gold disk electrode, the counter electrode was a piece of platinum wire and the reference electrode was a leakless Ag/AgCl electrode from eDAQ. Experiments were performed at room temperature. Solutions were purged with N_2_ before experiments, and an inert gas flux was left above the solution during experiments.

### Spectroelectrochemical experiments

Spectroelectrochemical experiments were performed with a Varian Cary Eclipse fluorescence spectrophotometer. Experiments were conducted with a 1 mm-optical path quartz cell from BAS Inc. using a gold mesh working electrode, a platinum counter electrode and a leakless Ag/AgCl reference electrode. The excitation wavelength was 543 nm and fluorescence spectra were recorded perpendicular to the excitation beam.

### *In situ* confocal laser scanning fluorescence microscopy

Fluorescence images were acquired at 20× magnification (Objective Leica HCX PL FLUOTAR, NA 0.4, working distance 6.9 mm) using a Leica SP5 DMI 6000B inverted microscope equipped with a 543 nm laser source and a spectral selector module in front of the photomultiplier tube detector. The default pinhole value was set at 106.1 μm corresponding to 1 Airy Unit. The laser power was in the range of 50 to 100% while the PMT gain was set at 700 V. The *in situ* microscopy experiments were conducted in a custom made glass cell with a 170 μm-thick glass optical window. As before, the reference electrode was a leakless Ag/AgCl (eDAQ) electrode, and the counter electrode was a piece of large area gold wire coiled around the working electrode. The working electrode was prepared with an optic fibre bundle (F&T Fibers and Technology GmbH, external diameter 350 μm, ≈20 000 fibres per mm^2^) following a previously reported procedure. Briefly, the fibre was carefully cut and polished to get a planar surface and further coated by sputtering a thin gold layer. The electrode surface was connected with silver glue and isolated with nail polish to leave only a cylindrical electrode surface area. The presence of the optic fiber array underneath the thin metal layer enables precise focusing at the electrode surface due to the observable pattern. Also, the thickness of the gold layer which is typically in the range of tenths of nm allows mitigation of reflection issues that are observed on bulk metal surfaces. To exclude possible artefacts (drift or positioning of the electrode), the series of fluorescence profiles were not collected with stepwise variation of the applied potentials but in a random fashion instead. Collected data were processed with LAS software from Leica. Fluorescence microscopy intensities are given in “arbitrary units” because the actual intensity does strongly depend on many parameters which cannot be kept constant (laser power, PMT gain, *etc.*) as the RZ and RF dyes exhibit rather different photo-physical properties.

## Conclusions

To conclude, mapping the fluorescence modulation in the vicinity of an electrode surface offers the possibility of deciphering the homogeneous chemical reactivity that is not observable when only recording electrochemical data. This was evidenced by recording *in situ* FCLSM that allows collecting images in the *x*–*y* plane at various *z*-distances from the electrode surface. When using fluorogenic species, such a technique allows probing the concentration profiles of the chemical species involved. As a proof-of-principle, a comproportionation reaction between RZ and DH, resulting in the formation of fluorescent RF with a maximum intensity located away from the electrode surface in the middle of the diffusion layer, was evidenced. This is indeed a very important result because nowadays RZ and RF dyes are commonly used for many fluorescence bioassays and such reactivity could potentially perturb the acquired fluorescence signal.

## Conflicts of interest

There are no conflicts to declare.

## Supplementary Material

Supplementary information
