# Supplementary material for: A snapshot of the electrochemical reaction layer by using 3 dimensionally resolved fluorescence mapping
Source: Chem Sci. 2018 Jul 16;9(32):6622–8. doi: 10.1039/c8sc02011f (PMC6115633; doi:10.1039/c8sc02011f)
Supplement: Supplementary file 1 [file SC-009-C8SC02011F-s001.pdf]

Electronic Supplementary Material (ESI) for Chemical Science.
This journal is © The Royal Society of Chemistry 2018

Snapshot Inside the Electrochemical Reaction Layer by Using 3 Dimensionally-Resolved Fluorescence Mapping

Anne de Poulpiquet,[a†] Bertrand Goudeau,[a] Patrick Garrigue,[a] Neso Sojic,[a] Stéphane Arbault,[a] Thomas Doneux *[b] and Laurent Bouffier*[a]

SUPPORTING INFORMATION

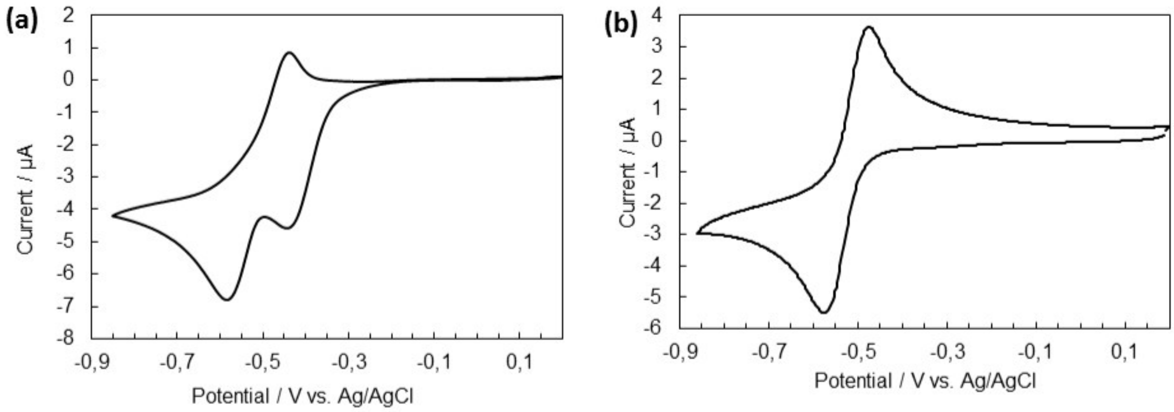

Figure S1. Cyclic voltammograms recorded at a Au disc electrode with a 1 mM (a) resazurin (RZ) or (b) resorufin (RF) solution. Experiments are performed in a 25 mM carbonate buffer solution (pH 10) at a scan rate of 10 mV s^{-1}. RZ is irreversibly reduced to the highly fluorescent RF according to a bielectronic reduction. RF is reversibly reduced to the not-fluorescent dihydroresorufin (DH) through another 2 electrons transfer.

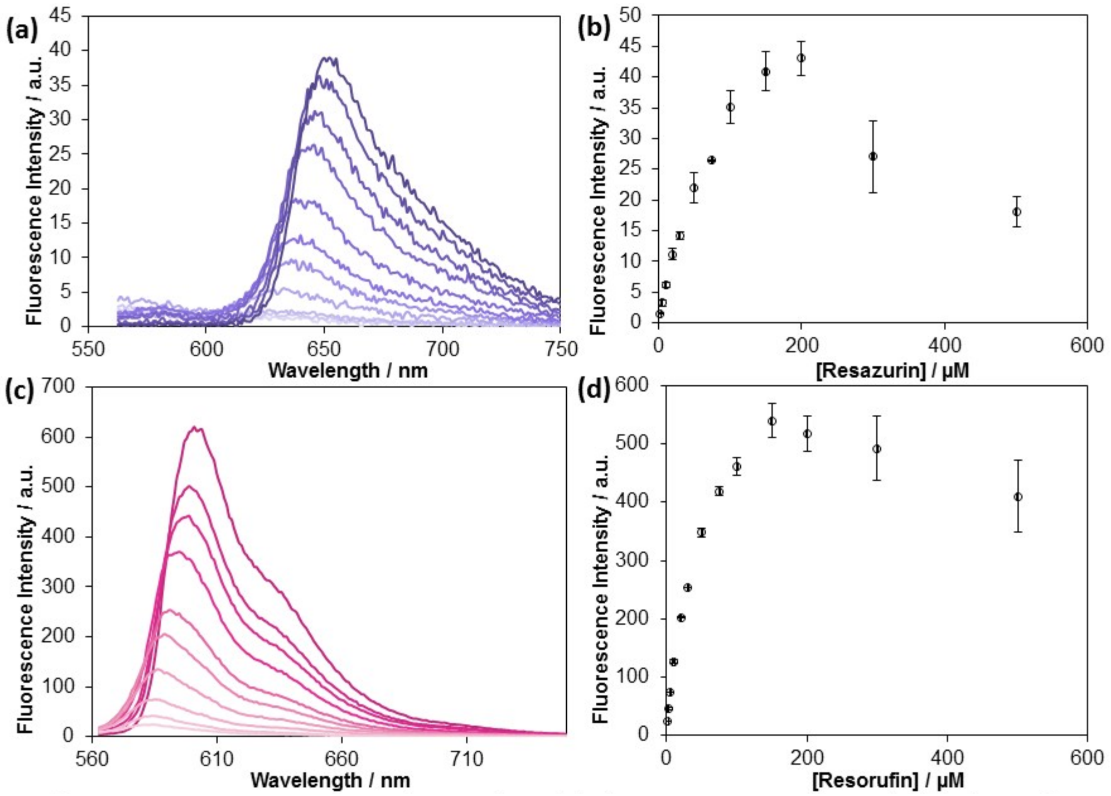

Figure S2. Fluorescence spectroscopy experiments. Fluorescence spectra recorded with increasing concentrations of (a) RZ or (c) RF. Corresponding plots of the maximal fluorescence intensity as a function of concentration of (b) RZ or (d) RF. Experiments were performed in 25 mM carbonate buffer solution (pH 10). $\lambda_{excitation}$ = 543 nm (± 2.5 nm), and fluorescence emission is collected perpendicular to the excitation beam.

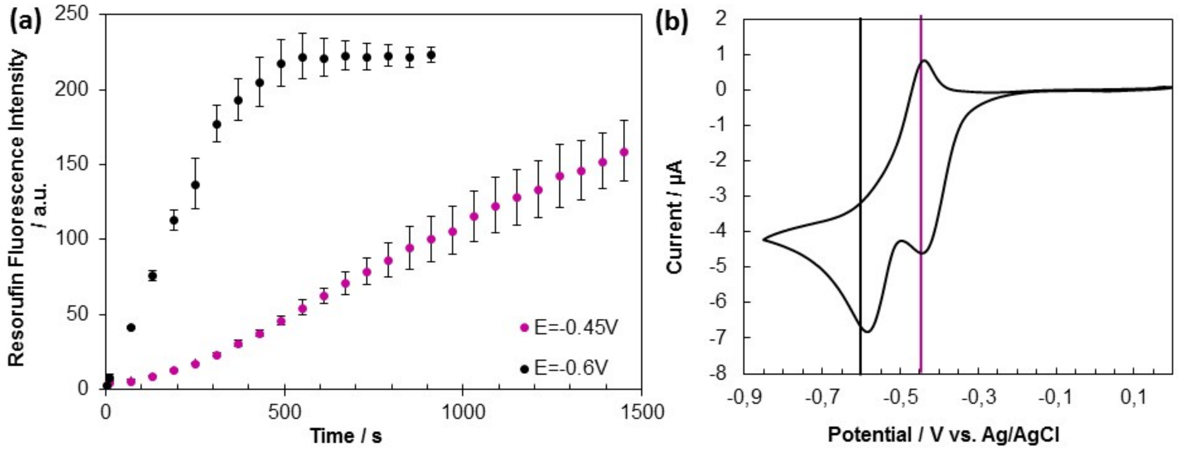

Figure S3. (a) Evolution of fluorescence intensity during RZ reduction at two different potentials, –0.45 V *vs*. Ag/AgCl (pink symbols) and –0.6 V (black symbols) observed by spectroelectrochemistry with a gold mesh working electrode, a platinum counter electrode and a leakless Ag/AgCl reference electrode. The excitation wavelength is 543 nm and fluorescence emission is collected perpendicular to the excitation beam at $\lambda = 587 \pm 2.5$ nm. Fluorescence spectra are collected at a regular interval time as soon as the potential is applied. (b) The two applied potentials are indicated in the CV to give an indication of their position relative to the characteristic redox potentials of the different species.

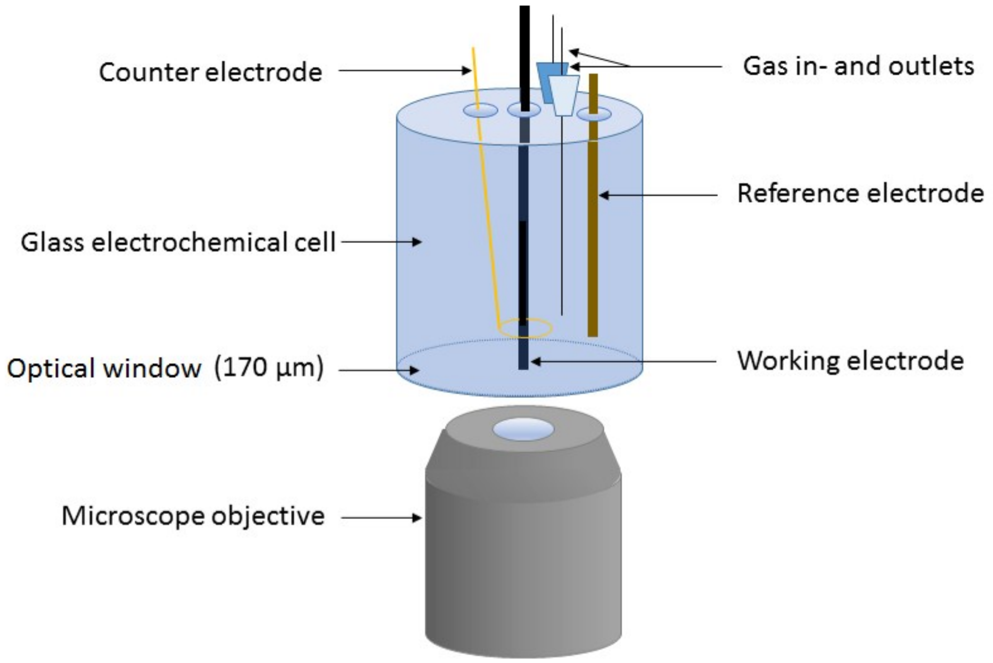

Scheme S1. Schematic representation of the experimental setup used in electrochemical confocal laser scanning microscopy (EC-CLSM). The electrochemical cell with a bottom made out of a transparent thin glass slide (170 µm-thick) is placed above the objective of an inverted epi-fluorescence microscope. The working electrode surface is a gold disc positioned in front of the objective. The reference electrode is a leakless Ag/AgCl reference electrode, and the counter electrode is a gold wired coiled around the working electrode. Both excitation laser beam and fluorescence collection occur through the objective.

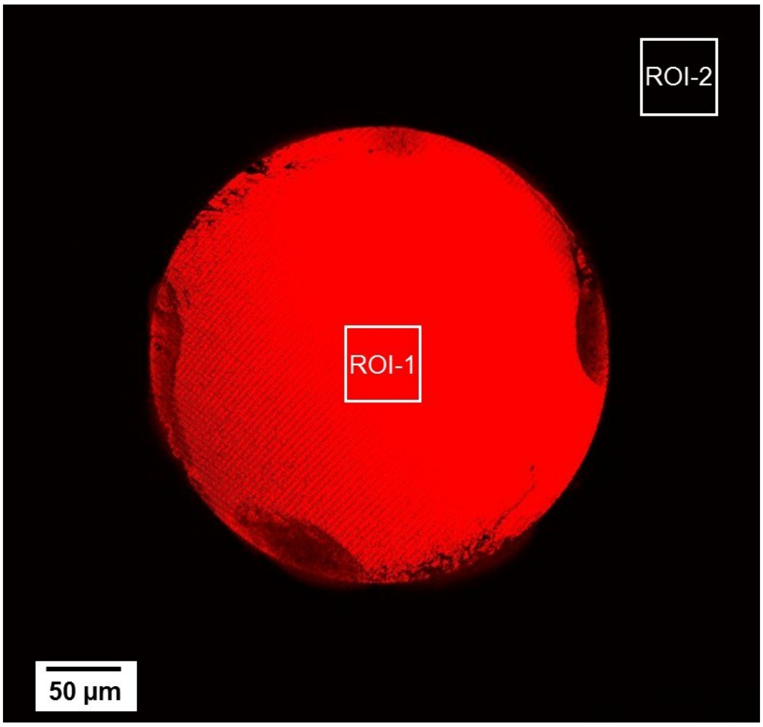

Figure S4. Typical image of the electrode recorded by using the reflection of the excitation laser showing the regions of interest (ROI) used for the analysis. ROI-1 is localized on the electrode whereas ROI-2 is further away from the electrode (i.e. in the bulk of the solution).

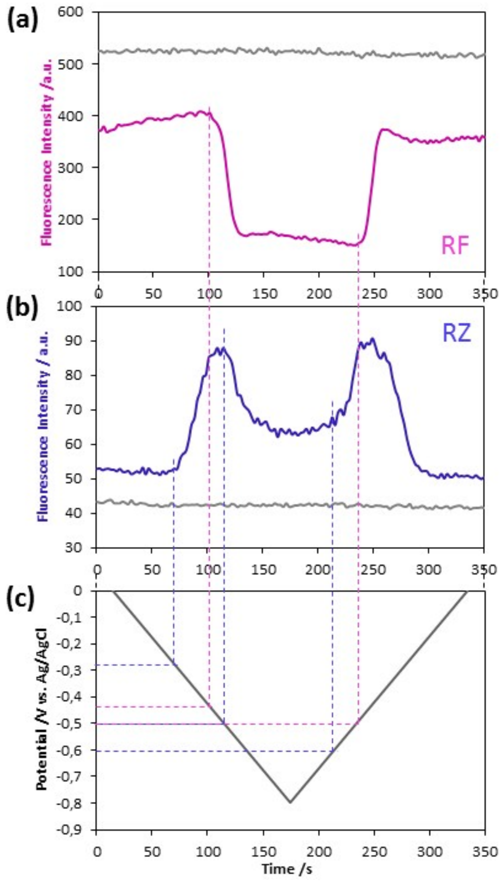

Figure S5. Evolution of the fluorescence intensity at the electrode surface (pink and blue curves) during the course of cyclic voltammetry with 100 µM resorufin (RF) solution (a) and 100 µM resazurin (RZ) solution (b). Grey lines indicate the variation of bulk fluorescence away from the electrode surface. (c) Corresponding time-evolution potential sweep. The dashed lines indicate the potentials of fluorescence onset/offset. Experiments performed in 25 mM carbonate buffer pH 10 degassed with Ar.

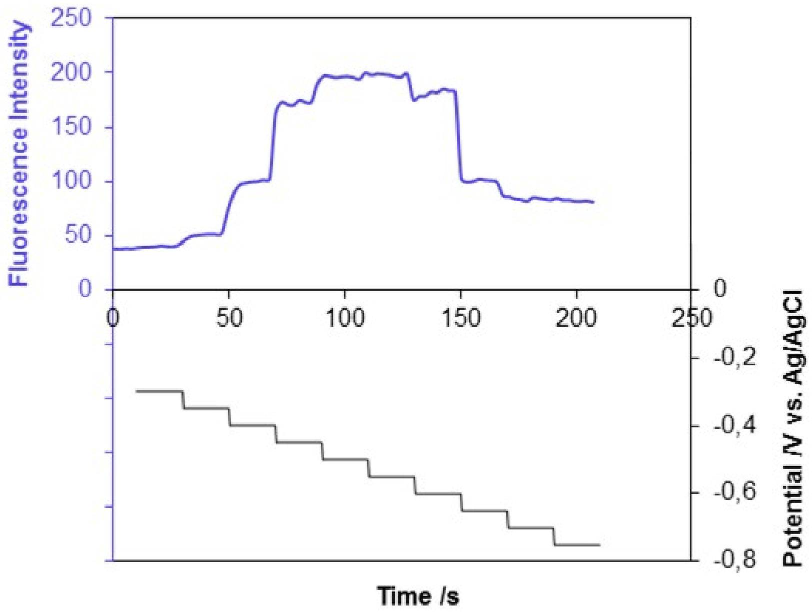

Figure S6. Evolution of the fluorescence intensity at the electrode surface (main axis) when successive potential steps are applied at the electrode in a 100 µM RZ solution. The successively applied potentials (*vs*. Ag/AgCl) are plotted against the secondary axis: –0.35 V ; –0.40 V ; –0.45 V ; –0.50 V ; –0.55 V ; –0.60 V ; –0.65 V ; –0.70 V ; –0.75 V ; –0.80 V. A potential of 0 V is applied before recording fluorescence. The experiment is performed in 25 mM carbonate buffer pH 10 degassed with Ar.

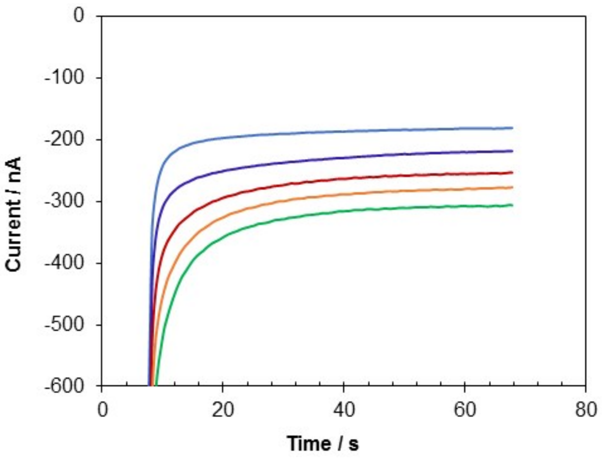

Figure S7. Current intensities recorded when different potentials (*vs.* Ag/AgCl) are applied at the electrode: –0.4 V (blue curve) ; –0.5 V (violet) ; –0.6 V (red) ; – 0.7 V (orange) and –0.8 V (green), in a 100 µM RF solution. Fluorescence profiles in Figure 3 are recorded during the corresponding potential steps.

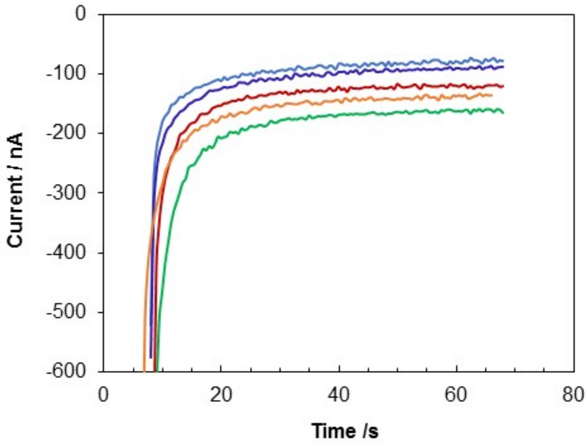

Figure S8. Current intensities recorded when different potentials (*vs.* Ag/AgCl) are applied at the electrode: –0.45 V (blue curve) ; –0.55 V (violet) ; –0.65 V (red) ; –0.75 V (orange) and –0.85 V (green), in a 100 µM RZ solution. Fluorescence profiles in Figure 2 are recorded during the corresponding potential steps.
